# The spillover effects of China’s carbon trading policy on coordinated emission efficiency

**DOI:** 10.1038/s41598-024-63157-x

**Published:** 2024-05-29

**Authors:** Hao Cui, Zengbing Liu

**Affiliations:** 1 College of Civil Engineering, Jiangxi Science and Technology Normal University, Nanchang, 330013 China; 2 Disaster Prevention and Mitigation Engineering Technology Research Base of Think Tank, Jiangxi Science and Technology Normal University, Nanchang, 330013 China

**Keywords:** Environmental sciences, Environmental social sciences

## Abstract

The significance of carbon trading policy (CTP) for China’s carbon reduction goals cannot be overstated. Examining the practical impacts and inherent features of this policy is crucial for fostering its healthy development and effectiveness. This study utilizes the directional SBM super-efficiency model to calculate the combined emission efficiency (CEE) of greenhouse gases and atmospheric pollutants across 30 provinces and cities in China from 2005 to 2020. Through spatiotemporal analysis of the CEE evolution using hotspot analysis, it is evident that hotspots gradually shift towards the southeast coastal areas over time, while cold spots shift towards the northwest. Additionally, employing the differences-in-differences (DID) model and conducting robustness tests, the study finds that the CTP significantly enhances the CEE development. Spatial econometric analysis reveals that the CEE primarily follows a distribution pattern characterized by low-low (LL) and high-high (HH) regions, with positive spatial spillover effects. However, due to the incomplete state of early green development in China, the CTP temporarily exhibits negative spatial spillover effects. Finally, considering the current state of China's carbon trading policy, corresponding policy recommendations are proposed in this paper.

## Introduction

Amidst global development, substantial amounts of greenhouse gases and pollutants continue to be emitted, presenting a significant threat to human progress, including issues such as global warming, rising sea levels, and extreme weather events^1,2^. China, as the world's second-largest economy, has experienced rapid economic and social growth, leading to increased emissions. However, the country is prioritizing green development as a core component of its national strategy, signaling a transition toward sustainable progress. As the top global carbon emitter, China has integrated low-carbon development into its long-term blueprint and introduced initiatives such as a pilot program for carbon emissions trading in several regions^3^. The program aims to trade carbon emission rights and increase the cost of emissions to drive reductions, reflecting China's commitment to mitigating its environmental impact.

Carbon trading policies have been widely acknowledged for their effectiveness in reducing carbon emissions. The Kyoto Protocol played a crucial role in initiating carbon trading markets, leading to significant reductions in air pollution and cost savings in air pollution management^4^. Additionally, studies have shown the EU's carbon emission trading system to effectively reduce regional carbon emissions^5^. Research by Chen et al.^6^ used global data analysis to validate the emission reduction impact of carbon trading policies in China, demonstrating improved carbon efficiency and reduced emissions. Gu et al.^7^ found that carbon emission reduction policies positively affect energy conservation and emissions reduction through statistical analysis. Furthermore, studies by Li^8^, Gao^9^, and Zhou^10^ collectively support the effectiveness of China's carbon trading policies in reducing regional or industry-specific carbon dioxide emissions.

Researchers have also explored the nexus between carbon trading policies and other regulations, as well as the regulatory function of carbon trading policies. Wang et al. employed the DID model and discerned substantial positive correlations between pilot carbon trading policies and e-commerce pilot city policies in mitigating carbon emissions^11^. Using a dynamic spatial econometric model and the system GMM estimation method, they revealed that policies such as carbon emission trading further augmented the efficacy of carbon emissions reduction in digital trade^12^.

Currently, academic research on carbon trading policies predominantly focuses on their efficacy in mitigating carbon emissions. However, the emission of carbon dioxide is intricately linked with that of other atmospheric pollutants, establishing an interconnection between greenhouse gas emissions and atmospheric pollutants. For instance, the combustion of fossil fuels not only produces atmospheric pollutants such as SO_2_, NO_x_, and particulate matter but also emits greenhouse gases like CO_2_^13^. Despite primarily addressing carbon emissions, carbon trading also harbors the potential to alleviate the release of other atmospheric pollutants. Both Burtraw et al. and Thompson et al. have argued that policies aimed at reducing greenhouse gases can also result in a reduction in air pollutants^14,15^.

In terms of research objectives, the study can be broadly categorized into two perspectives: the single-factor perspective and the total-factor perspective. The single-factor perspective is delineated by carbon productivity, computed as the gross domestic product (GDP) estimated by carbon emissions^16^. However, this approach neglects the contributions of various other influencing factors. Therefore, researchers^17–19^ have advocated for a total-factor perspective to assess carbon emissions comprehensively. Stochastic frontier analysis (SFA) is suited only for a single output, posing challenges in handling unexpected outputs^20^. The Non-parametric DEA is another method employed to gauge carbon emissions efficiency through linear programming. Nonetheless, the traditional DEA models encounter issues concerning input–output radial and further comparative difficulties among active decision-making units^21,22^. To tackle these challenges, several enhanced DEA models have been proposed and implemented in empirical research.

Tone proposed the SBM-DEA model^23^, subsequently utilized by Iftikhar et al. for both static and dynamic studies on the energy and CO_2_ emission efficiency of major economies^24^. Expanding upon this work, Tone introduced the SE-SBM model^25^, while Xie et al. applied the super-SBM model to assess the carbon emission efficiency of 59 countries between 1998 and 2016^26^. Their findings revealed a consistent improvement in China's carbon emission efficiency, albeit with some fluctuations, and underscored significant potential for enhancing global carbon emission efficiency. Furthermore, Oh^27^ introduced the global Malmquist-Luenberger index, and Wang et al. examined the static and dynamic efficiency of carbon emissions among 23 Chinese airlines from 2009 to 2013^28^. Their analysis utilized the global slack-based measure (GSBM) model alongside the global Malmquist-Luenberger (GML) index, uncovering various factors contributing to dynamic efficiency changes among airlines. Additionally, Toshiyuki et al. presented the intermediate DEA model^29^, utilized by Sun et al. to compute the unified efficiency of provincial fossil fuel power plants in China from 2005 to 2015^30^. Finally, Cheng et al. devised a novel meta-frontier total-factor carbon emission efficiency (TCEI) index using an improved non-radial directional distance function^31^. They estimated the meta-frontier TCEI for 30 provincial industrial sectors in China from 2005 to 2015 and analyzed its dynamic evolution. Interestingly, their study demonstrated that the improved NDDF offered greater advantages in measuring carbon emission efficiency and technological gaps compared to the traditional NDDF.

This study adopts a total-factor perspective to address the research topic, integrating labor, fixed asset investment, and energy consumption as inputs, with GDP as the desired output, and carbon dioxide and sulfur dioxide as undesired outputs. This comprehensive approach encompasses both greenhouse gases and atmospheric pollutants in the analysis. Moreover, the study utilizes the Directional SBM super-efficiency model^32^ to calculate the CEE of 30 provinces and cities in China, exploring the distribution of the CEE hotspots. Additionally, leveraging panel data from 30 provinces and cities in China, the research investigates the impact of carbon trading policies on the CEE using the DID model. Finally, spatial econometric models are employed to analyze the spatial spillover effects of the CEE and carbon trading policies.

## Research methods and data sources

### Research methods

#### Data envelopment analysis

The DEA method assesses relative efficiency by comparing decision-making units' inputs and outputs through linear programming. The Directional SBM super-efficiency model, a variant of DEA, considers the direction of inputs and outputs in production, enhancing accuracy in reflecting real production processes. It offers greater flexibility and adaptability to diverse production settings, especially in handling incomplete data. Additionally, this model incorporates the decision unit's elastic range, ensuring stability in efficiency evaluation despite minor fluctuations. Unlike traditional models, the Directional SBM super-efficiency model is robust in dealing with uncertainties and variations in practical production scenarios. Building on Fukuyama et al.'s work^32^, the Directional SBM super-efficiency model was used to assess the Carbon Emission Efficiency (CEE).

#### Hotspot analysis

Hotspot analysis facilitates the identification of statistically significant high or low values, thereby exploring the spatial distribution characteristics of the observed objects. By examining hotspots, it can aid in predicting potential future trends and offering decision-making insights for governments, enterprises, and individuals. Consequently, this paper investigates the distribution trend of the CEE in China through hotspot analysis.

1 $$G_{i}^{*} = \frac{{\sum\limits_{j = 1}^{n} {\omega_{i,j} x_{j} - \overline{X}\sum\limits_{j = 1}^{n} {\omega_{i,j} } } }}{{S\sqrt {\frac{{n\sum\limits_{j}^{n} {\omega_{i,j}^{2} - (\sum\limits_{j = 1}^{n} {\omega_{i,j} } )^{2} } }}{n - 1}} }},$$

2 $$\overline{X} = \frac{{\sum\limits_{j = 1}^{n} {x_{j} } }}{n},$$

3 $$S = \sqrt {\frac{{\sum\limits_{j = 1}^{n} {x_{j}^{2} } }}{n} - (\overline{X})^{2} } ,$$

where *x*_*j*_ refers to the CEE value of region *j*, while $\omega_{i,j}$ represents the spatial weight between regions *i* and *j*, and *n* stands for the total number of regions. A significantly positive value for $G_{i}^{*}$ identifies city *i* as a hotspot area for building green land use efficiency, whereas a non-significant or negative value categorizes it as a cold spot or insignificant area.

#### DID model

The DID model is a commonly used econometric technique for assessing the effects of policies on a target group. By comparing changes before and after policy implementation between experimental and control groups, the DID approach effectively addresses endogeneity and selection biases, improving result credibility. This method controls for time-invariant factors and individual differences, enhancing the accuracy of policy impact evaluations. Unlike traditional models, DID accounts for time trends and individual variations, strengthening research reliability and validity.

4 $$Y_{it} = \beta_{0} + \beta_{1} du \times dt + \lambda_{i} + v_{t} + \varepsilon_{it} ,$$

In the equation, d*u* represents a grouping dummy variable. If individual *i* is affected by policy implementation, *i* belongs to the treatment group, and the corresponding value of d*u* is 1. If individual *i* is not affected by policy implementation, *i* belongs to the control group, and the corresponding value of d*u* is 0. d*t* represents a policy implementation dummy variable. Before policy implementation, d*t* has a value of 0, and after policy implementation, d*t* has a value of 1. d*u*×d*t* represents the interaction term between the grouping dummy variable and the policy implementation dummy variable. The coefficient of d*u*×d*t* reflects the net effect of policy implementation.$\lambda_{i}$ represents individual fixed effects, accurately reflecting individual characteristics.$v_{t}$ represents time fixed effects, accurately reflecting time characteristics.$\varepsilon_{it}$ represents the disturbance term.

#### Moran’s I

Moran's I is a widely used spatial autocorrelation index employed for assessing spatial autocorrelation, identifying spatial clustering and spatial heterogeneity, and providing spatial analysis. Its application has been extensive, finding wide usage in various domains including Geographic Information Science, sociology, economics, among others.

5 $$I = \frac{n}{{S_{o} }}\frac{{n\sum\limits_{i = 1}^{n} {\sum\limits_{j = 1}^{n} {\omega_{i,j} z_{i} z_{j} } } }}{{\sum\limits_{i = 1}^{n} {z_{i}^{2} } }},$$

6 $$S_{o} = \sum\limits_{i = 1}^{n} {\sum\limits_{j = 1}^{n} {\omega_{i,j} } } ,$$

7 $$I_{i} = \frac{{Z_{i} }}{{\sum\limits_{i = 1}^{n} {z_{i}^{2} } }}\sum\limits_{j}^{n} {\omega_{i,j} Z_{i} } ,$$

where *I* stands for the global Moran's *I*, *Ii* represents the local Moran's *I*, *z*_*i*_ signifies the deviation of region *i*'s CEE value from its mean, and $\omega_{i,j}$ is the spatial weight between region *i* and region* j*. The variable *n* denotes the total number of regions, and *So* signifies the aggregation of all spatial weights (Neighboring areas are denoted as 1, while non-neighboring areas are denoted as 0).

#### The spatial Durbin model

The Spatial Durbin Model (SDM) serves as an econometric tool to analyze spatial spillover effects, adept at capturing interregional interactions and dependencies critical for comprehending regional dynamics. The SDM's versatility extends to analyzing diverse spatial datasets and research inquiries, such as inter-regional economic relationships and intercity policy impacts. Therefore, this research leverages the SDM to explore the spatial spillover impact of the Carbon Trading Policy (CTP) on Carbon Emission Efficiency (CEE).

8 $$y_{it} = \rho \sum\limits_{j = 1}^{N} {W_{ij} } y_{jt} + \partial x_{it} + \theta \sum\limits_{j = 1}^{N} {W_{ij} x_{jt} + \varepsilon_{it} } ,$$

In the formula, *y* signifies the CEE. *x* represents the explanatory variable. $\rho$ and $\theta$ indicate spatial autocorrelation coefficients for the CEE and the explanatory variable, respectively. *W* is the spatial weight matrix.$\partial$ denotes the regression coefficient of the explanatory variable.$\varepsilon$ represents the error term. *i* and *j* signify the regions, and *t* stands for time. The variable *N* corresponds to the total number of provinces.

### Variable description

Table 1 presents the acronyms of the variables examined in this study. The year preceding the implementation of the Carbon Trading Policy (CTP) is denoted as 0, while the implementation year and the subsequent year are denoted as 1. Districts without CTP implementation are labeled as 0, while those with CTP implementation are labeled as 1. The interaction term between districts and years quantifies the CTP variable. We compute the Per capita gross domestic product (PGDP) by dividing GDP by the population of each province, following the methodology outlined by Ji et al.^33^. Capital misallocation (CM) is calculated using the approach established by Ji et al.^33^. Industrial structure (IS) is determined as the ratio of the tertiary industry's added value to GDP, while Openness (OP) is derived by dividing the total foreign investment by GDP. Similarly, Energy intensity (EI) is obtained by dividing the total energy consumption by GDP. Green innovation (GI) is measured using the number of green patent applications, and technological activity (TA) is determined by dividing the value of technological contract transactions by GDP. To compute GFI, we employ the entropy weight method as recommended by Yu et al.^34^ and Fang et al.^35^.

**Table 1:** Variable codes.

Variable	Acronyms
Dependent variable	
Cooperative emission efficiency	CEE
Core explanatory variable	
Carbon trading policy	CTP
Control variable	
Per capita gross domestic product^36^	PGDP
Capital misallocation^37^	CM
Industrial structure^38^	IS
Openness^39^	OP
Energy intensity^40^	EI
Green innovation^41^	GI
Technological activity^42^	TA
Green finance index^43^	GFI

### Data sources

The data on carbon dioxide (CO_2_) and sulfur dioxide (SO_2_) emissions utilized in this study were sourced from the multi-resolution emission inventory model for climate and air pollution research^44–47^. Additionally, control variable data were gathered from authoritative institutions, including the National Bureau of Statistics, the Ministry of Science and Technology, the People's Bank of China, and various reputable statistical yearbooks. These sources encompass national and provincial statistical yearbooks, environmental status bulletins, and specialized statistical yearbooks such as the "China Science and Technology Statistical Yearbook", "China Energy Statistical Yearbook", "China Financial Yearbook", "China Agriculture Statistical Yearbook", "China Industry Statistical Yearbook," and "China Tertiary Industry Statistical Yearbook."

## Results and discussion

### Analysis of spatiotemporal evolution characteristics of CEE

**Figure 1:**
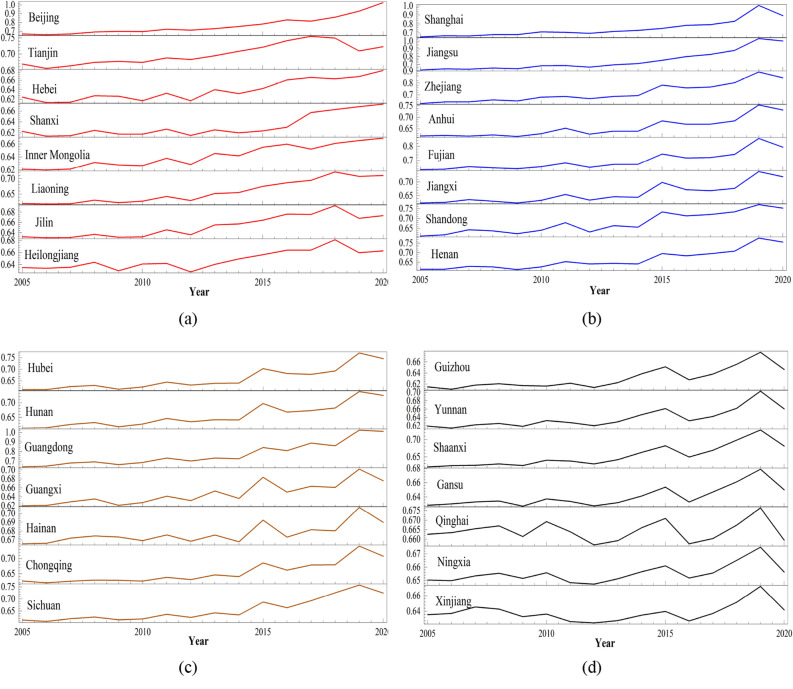
Changes in the CEE over time by province in China from 2005 to 2020.

**Figure 2:**
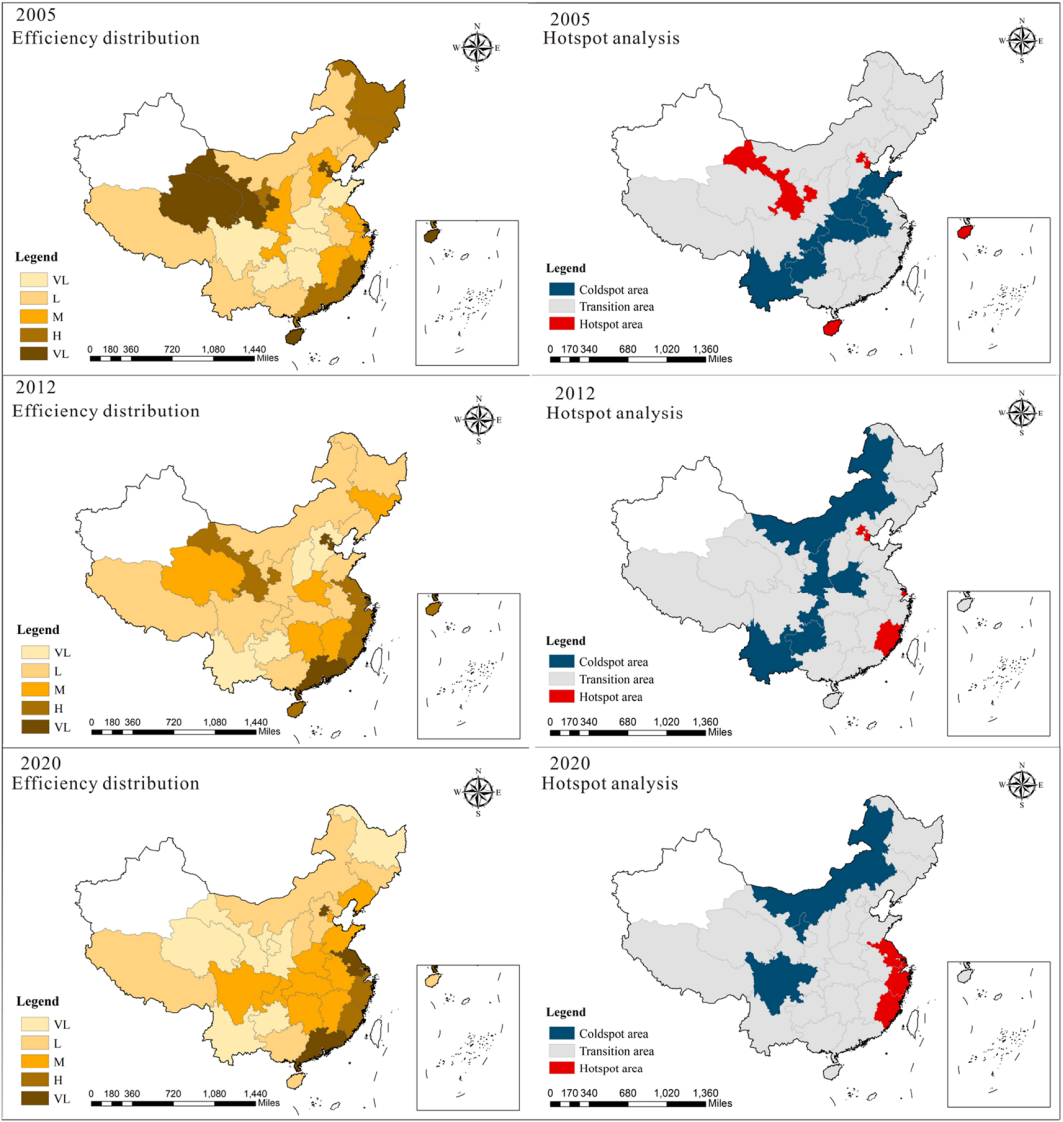
Spatial distribution and hotspot analysis of CEE (drawn by ArcMap 10.8.1, http://www.arcgis.com).

The Directional SBM super-efficiency model and hotspot analysis were employed to depict the CEE across Chinese provinces over various years, as detailed in Fig. 1. Additionally, Fig. 2 illustrates the spatial evolution of the CEE in China for 2005, 2012, and 2020. This study adopts the natural breakpoint classification method to stratify the CEE into five levels: very low (VL), low (L), medium (M), high (H), and very high (VH). Hotspot analysis further classifies areas into cold spots, transitional zones, and hot spots. The spatial distribution of China's CEE exhibits significant spatial heterogeneity. Analysis of the temporal and spatial characteristics reveals that the advantage of the CEE in eastern cities was less prominent in earlier years, with the CEE hotspots being relatively dispersed. However, over time, the CEE hotspots gradually shifted towards southeastern coastal regions, while the CEE cold spots progressed inland. Hotspots are mainly concentrated in regions such as Jiangsu, Zhejiang, Shanghai, and the Beijing-Tianjin-Hebei area.

The limited progress of green development in the eastern region during its initial phase can be attributed to various factors. Firstly, the region's industrial landscape was predominantly characterized by traditional sectors like heavy chemicals, steel, and coal, known for their high energy consumption and pollution. This lack of environmental focus led to substantial pollution problems. Additionally, the eastern region's abundant economic, human, and natural resources were extensively exploited, causing overconsumption and environmental harm. Both government and enterprises prioritized economic growth over environmental concerns, resulting in insufficient investment in environmental protection. Furthermore, the area's developed economy and commercial interests led some businesses to prioritize short-term gains by pursuing polluting industries. Finally, inadequate technology and management practices hindered pollution control efforts.

Nevertheless, spurred by growing environmental awareness and guided by governmental policies, the eastern region has progressively recognized the significance of green development. It has enacted a spectrum of measures aimed at enhancing the ecological landscape. These initiatives encompass bolstering environmental legislation, fostering the refinement and modernization of industrial frameworks, propelling the advancement and adoption of clean energy sources, and intensifying environmental oversight. Consequently, the environmental integrity of the eastern region has steadily ameliorated, laying the foundation for the realization of sustainable development.

### Analysis of the role of CTP in CEE

#### Parallel trend test

**Figure 3:**
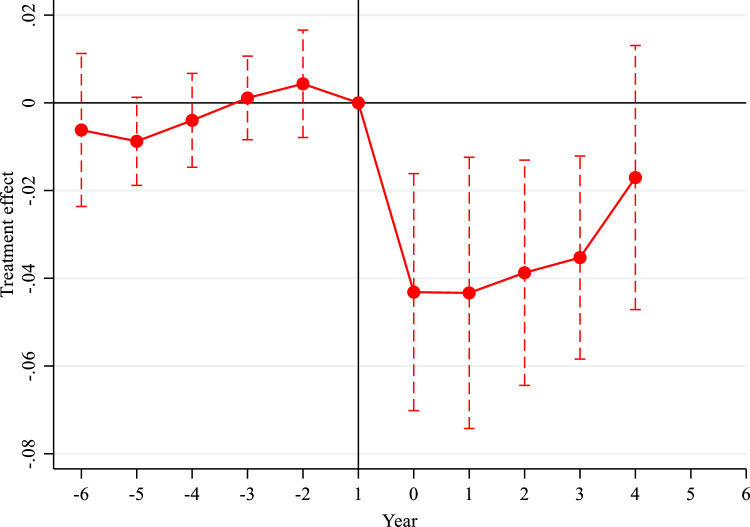
Results of parallel trend test.

Given its status as a key policy in China's carbon emission reduction efforts, it is crucial to assess the positive impact of the CTP on the CEE. This paper employs the DID model to explore the influence of the CTP on the CEE. Initially, a parallel trend test was conducted using panel data from 30 provinces and cities in China from 2005 to 2016, with the pilot year of the CTP as the baseline, as depicted in Fig. 3. Prior to the implementation of the CTP, when the year is less than 0, the estimated coefficient's 95% confidence interval contains 0, indicating that the influence of the CTP is not significant. However, following the implementation of the CTP, its impact becomes statistically significant, with a negative coefficient. The absolute value of the coefficient diminishes over time. This can be attributed to the initial stage of the CTP implementation, during which enterprises increase investment in technological and operational costs, leading to a temporary suppression of the CEE. However, over time, this suppressive effect diminishes. From a long-term perspective, the CTP is generally beneficial for enhancing the CEE. In conclusion, there was no significant difference in the changes in the CEE between the treatment group and the control group prior to the implementation of the CTP, supporting the applicability of the DID model to evaluate the treatment effect of the CTP.

#### The results of benchmark regression

This study employs the DID model to estimate the impact of the CTP on the CEE of Chinese provinces and cities, and the results are presented in Table 2. Prior to the introduction of control variables, the estimated coefficient for the CTP is positive, indicating an enhancement of the CEE due to the CTP at a significance level of 1%. With the introduction of control variables, the sign of the estimated coefficient remains unchanged, retaining significance at a level of 5%. Overall, the CTP has improved the CEE in China. On one hand, the CTP can encourage green innovation, promote the development of sustainable technologies, and achieve emissions reduction either directly or indirectly by increasing the cost of carbon emissions. Additionally, the carbon trading policy can put pressure on companies to implement resource conservation and recycling during production processes by making them aware of the cost of carbon emissions^48^. On the other hand, the CTP restricts greenhouse gas emissions and issues corresponding emission allowances, providing low-carbon technologies and investments with economic incentives and encouraging corporate transition towards low-carbon, clean energy, and sustainable development.

**Table 2:** The results of benchmark regression.

CEE	Coefficient	Robust std. err	P	Coefficient	Robust std. err	P
CTP	0.0283	0.0102	0.010	0.0212	0.0092	0.029
Cons	0.6310	0.0032	0.000	1.0389	0.1962	0.000
Control	NO	NO	NO	Yes	Yes	Yes
Province FE	Yes	Yes	Yes	Yes	Yes	Yes
Year FE	Yes	Yes	Yes	Yes	Yes	Yes

#### Placebo test

**Figure 4:**
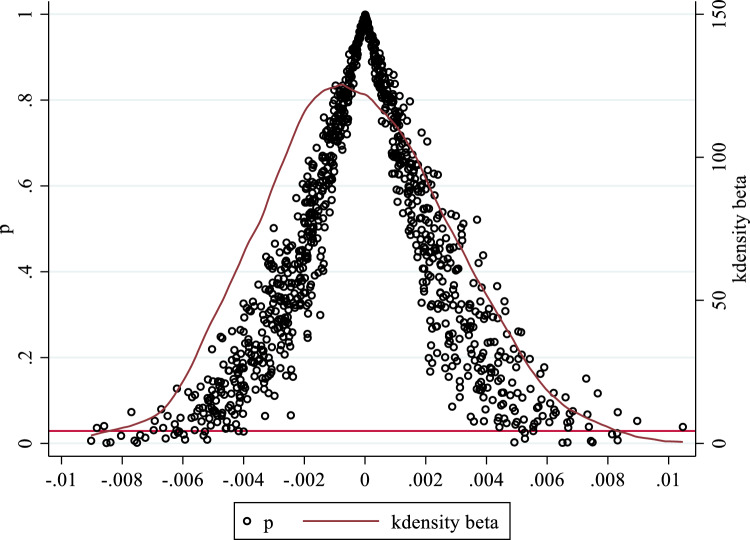
Placebo test results.

To further mitigate any potential influence of unobserved confounding factors, we conducted a placebo test using 1000 random samples. In each iteration, 72 samples were designated as the treatment group, while the remainder served as the control group for regression analysis. As depicted in Fig. 4, we visualize the distribution of 1000 estimated coefficients and their corresponding p-values. The X-axis represents the magnitude of the estimated coefficients, while the Y-axis displays the density value and p-value. The curve illustrates the kernel density distribution of the estimated coefficients, with black dots denoting the associated p-values for each coefficient. The red horizontal line represents the true estimated value of 0.029 for the CTP from the DID model. Our analysis reveals that the majority of estimated coefficients cluster around zero, with p-values for most estimates exceeding 0.1, thus lacking statistical significance at the 10% level. This suggests that our estimated outcomes are improbable to occur by chance and are unlikely to be influenced by other policies or random factors. Consequently, we confidently assert that the placebo test was effective, and the positive impact of the CTP on carbon emissions intensity is not a random occurrence.

#### Robustness test

To ensure the reliability of the DID model findings while considering fixed time and individual effects, this study initiates regression analyses utilizing emission efficiencies calculated solely for sulfur dioxide as a non-desired output (SDEE) and emission efficiencies calculated solely for carbon dioxide as a non-desired output (CDEE) as surrogate dependent variables. Subsequently, to mitigate endogeneity concerns, lag 1, lag 2, and lag 3 cycles of the CTP are incorporated into the DID model for regression analysis.

After substituting the CEE with the SDEE and the CDEE, the regression results presented in Table 3 were obtained. At a significance level of 1%, the coefficient of the CTP is positively significant in the regression with the SDEE, while at a significance level of 5%, the coefficient of the CTP is also positively significant in the regression with the CEE. These findings demonstrate the robustness of the DID model following the replacement of the dependent variable.

**Table 3:** Regression results with replacement of similar dependent variables.

	SDEE	CDEE
	Coefficient	Coefficient
CTP	0.0215*** (0.007)	0.0208** (0.010)
PGDP	− 0.0825** (0.040)	− 0.0594 (0.044)
IS	− 0.0173 (0.076)	− 0.0341 (0.076)
OP	0.0008 (0.001)	0.0013 (0.002)
EI	− 3.59e–06 (0.001)	0.0001 (0.001)
GI	0.0148 (0.018)	0.0125 (0.018)
TA	0.4697** (0.222)	− 0.0642 (0.232)
CM	− 0.0243 (0.023)	− 0.0148 (0.025)
GFI	0.0176 (0.047)	0.0126 (0.070)
Cons	0.9630***	0.8838***
Province FE	Yes	Yes
Year FE	Yes	Yes

*** represents P < 0.01, ** represents 0.01 < P < 0.05, and the numbers in parentheses represent the robust standard error.

By introducing the lagged 1, lagged 2, and lagged 3 periods of the CTP into the DID model for regression analysis, the findings reported in Table 4 were produced. At a significance level of 1%, the coefficients for the lagged 1 and lagged 3 periods of the CTP are positive, whereas at a significance level of 5%, the coefficient for lagged 2 period of the CTP is also positive. These findings suggest that the DID model remains robust after considering some of the endogeneity.

**Table 4:** Regression results with lagged periods.

Coefficient	CEE	CEE	CEE
L.CTP	0.0212*** (0.008)		
L2.CTP		0.0226** (0.009)	
L3.CTP			0.0258*** (0.009)
PGDP	− 0.1156*** (0.042)	− 0.1152** (0.042)	− 0.1019** (0.049)
IS	− 0.0773 (0.077)	− 0.0989 (0.081)	− 0.1267 (0.085)
OP	0.0017 (0.001)	0.0024* (0.001)	0.0026 (0.002)
EI	0.0001 (0.001)	0.0001 (0.001)	0.0001 (0.001)
GI	0.0146 (0.015)	0.0107 (0.015)	− 0.0011 (0.014)
TA	0.2328 (0.246)	0.1623 (0.254)	0.0530 (0.267)
CM	− 0.0112 (0.023)	− 0.0154 (0.019)	− 0.0171 (0.019)
GFI	0.0679 (0.055)	0.0893 (0.059)	0.0723 (0.059)
Cons	1.0782***	1.0938***	1.0989***
Province FE	Yes	Yes	Yes
Year FE	Yes	Yes	Yes

*** represents P < 0.01, ** represents 0.01 < P < 0.05, * represents 0.05 < P < 0.1, and the numbers in parentheses represent the robust standard error.

### Spatial characteristics analysis

#### Spatial autocorrelation analysis of CEE

The hotspot analysis reveals a noticeable spatial correlation within the CEE. Therefore, this study investigates the spatial correlation of the CEE using Moran's I index. The selected weight matrix is a neighborhood matrix (0–1), and the final result is shown in Table 5. The Moran's I index of the CEE consistently exhibits positivity from 2005 to 2020, with a majority being statistically significant at the 1% threshold. These results indicate a positive spatial correlation and the presence of spatial spillover effects in the CEE.

**Table 5:** Global Mora’s I index.

Year	I	Sd(I)	z	P-value	Year	I	Sd(I)	z	P-value
2005	0.328	0.123	2.958	0.003	2013	0.398	0.121	3.579	0.000
2006	0.364	0.124	3.215	0.001	2014	0.323	0.120	2.979	0.003
2007	0.378	0.124	3.330	0.001	2015	0.371	0.120	3.384	0.001
2008	0.385	0.122	3.431	0.001	2016	0.400	0.121	3.584	0.000
2009	0.396	0.121	3.551	0.000	2017	0.283	0.118	2.683	0.007
2010	0.415	0.122	3.671	0.000	2018	0.286	0.121	2.660	0.008
2011	0.428	0.121	3.816	0.000	2019	0.375	0.120	3.409	0.001
2012	0.359	0.121	3.255	0.001	2020	0.239	0.118	2.310	0.021

To gain further insight into the spatial distribution of China's provincial CEE, this study employs Moran scatter plots to illustrate its spatial agglomeration. The four quadrants of the Moran scatter plot denote spatial clusters of low–high (LH), high-high (HH), high-low (HL), and low-low (LL), arranged clockwise from the upper-left quadrant. The origin of the Moran scatter plot represents the global Moran's I index for that year, while the distance between sample points and the origin indicates the level of significant agglomeration, with greater distances signifying higher significance.

**Figure 5:**
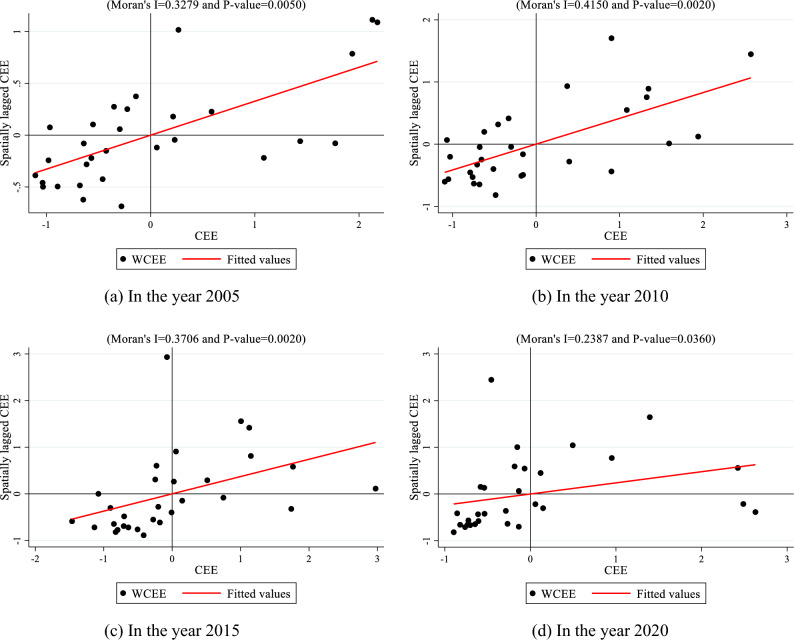
Moran scatter plots of the CEE in China for the years 2005, 2010, 2015, and 2020. (**a**) In the year 2005, (**b**) In the year 2010, (**c**) In the year 2015, (**d**) In the year 2020.

As depicted in Fig. 5, the Moran scatter plot of the CEE for 2005, 2010, 2015, and 2020 predominantly falls within the third and first quadrants, indicating clustering of China's CEE primarily in LL and HH regions. However, HH cluster exhibits greater significance compared to LL cluster. The majority of HH clustered areas are situated in the eastern coastal provinces and cities, such as Beijing, Tianjin, Shanghai, Zhejiang, Jiangsu, and Guangdong. This phenomenon arises from multifaceted interactions and mutual influences. Firstly, the eastern coastal provinces and cities benefit from superior geographical positioning, facilitating closer ties to international trade and oceanic resources. This proximity enables enhanced access to international markets, technology, and financial support, consequently fostering green development. Secondly, these regions generally boast higher levels of economic development, providing a robust foundation for green initiatives. Greater economic development translates to increased investment and resources allocated to environmental protection and the advancement of green technologies. Additionally, the concentration of innovative enterprises and higher education institutions in these regions furnishes superior human resources support for green technology innovation and application. This concentration fosters technology exchange and cooperation, thereby expediting green development. Lastly, government policy support and planning guidance for green development play pivotal roles in clustering green CEE in HH region of eastern coastal provinces and cities. These areas often implement specific, targeted policies and plans promoting green industry development, resource conservation and utilization, and environmental protection, thus contributing to HH clustering of green CEE.

#### Analysis of spatial spillover effects of CTP

The Moran's Index confirms the presence of spatial spillover effects in the CEE**.** This raises the question of whether the CTP also demonstrates spatial spillover effects and significantly influences the CEE in the surrounding areas of the pilot provinces and cities. This study investigates the spatial spillover effects of the CTP using a spatial autoregressive model. The outcomes from Table 6 reveal that both the LM test and Robust-LM test demonstrate statistically significant results for the Spatial Error Model (SEM), whereas the spatial lag model (SLM) yields nonsignificant findings. Consequently, the SEM is considered more suitable for this study than the SLM.

**Table 6:** The results of LM test and robust-LM test.

Test	Statistic	df	P-value
Spatial error			
Moran’s I	3.245	1	0.001
LM	170.596	1	0.000
Robust-LM	171.951	1	0.000
Spatial lag			
LM	0.083	1	0.773
Robust-LM	1.438	1	0.230

However, the SEM does not directly explain the spatial spillover effects of the independent variables. Therefore, this study considers adopting the SDM. Firstly, Wald and LR tests were conducted, detailed in Table 7, showing positive and statistically significant results, indicating that the SDM does not collapse into the SLM or SEM. With a positive statistic in Hausman test and significant results, fixed effects are deemed more suitable for this study. Subsequently, through the LR test, both positive statistics with significant results lead to the selection of double fixed effects. Thus, this study utilizes the SDM with time and individual double fixed effects to analyze the spatial spillover effect of the CTP.

**Table 7:** The results of Waldtest, Hausmantest and LR test.

Test		Statistic	P-value
Wald	Test for SLM	42.28	0.000
	Test for SEM	32.81	0.001
LR	LR test SDM SLM	58.99	0.000
	LR test SDM SEM	41.72	0.000
Hausman		184.12	0.000
LR	LR test both ind	46.98	0.019
	LR test both time	170.38	0.000

#### Analysis of regression results

The SDM decomposes the effect of Carbon Trading Pilot on the CEE into direct and indirect components. The direct effect represents the influence of a province's CTP on its own CEE. The indirect effect comprises two distinct elements: one is the impact of a province's CTP on the CEE of neighboring provinces, and the other is the feedback loop effect, where a province's CTP affects its own CEE, subsequently influencing the CEE of neighboring provinces.

As indicated in Table 8, both the overall effect and the direct effect of the CTP on the CEE exhibit positive impacts at the 1% level of significance. This finding further corroborates the results obtained from the DID model. Additionally, rho in Table 8 also demonstrates a significant positive effect at the 1% level of significance, suggesting that the advancement of the CEE itself contributes to the enhancement of the CEE in neighboring regions, thereby indicating a positive spatial spillover effect.

**Table 8:** The regression results of the SDM.

CEE	Benchmark regression	Main	LR_Direct	LR_Indirect
CTP	0.0212** (0.009)	0.0401*** (0.070)	0.0341*** (0.007)	− 0.0692*** (0.024)
PGDP	− 0.1088** (0.044)	0.0719** (0.033)	0.0451 (0.030)	− 0.2934*** (0.065)
IS	− 0.0437 (0.080)	− 0.003 (0.042)	− 0.0013 (0.043)	− 0.0102 (0.127)
OP	0.0009 (0.002)	0.0081** (0.004)	0.0052 (0.004)	− 0.0314 (0.023)
EI	0.0001 (0.001)	0.0004 (0.001)	0.0005 (0.001)	0.0010 (0.001)
GI	0.0193 (0.017)	− 0.0270** (0.013)	− 0.0204 (0.013)	0.0663** (0.031)
TA	0.1996 (0.241)	0.7932*** (0.190)	0.6421*** (0.211)	− 10.7351** (0.749)
CM	− 0.0098 (0.025)	− 0.0051 (0.014)	0.0043 (0.016)	0.1149* (0.063)
GFI	0.0371 (0..054)	0.0389 (0.059)	0.0940 (0.063)	0.5852*** (0.187)
Spatial				
rho		0.5230*** (0.039)		

*** represents P < 0.01, ** represents0.01 < P < 0.05, * represents 0.05 < P < 0.1, and the numbers in parentheses represent the standard error.

The indirect effect of carbon trading policy on the CEE demonstrates a significant negative influence at the 1% level. This phenomenon can be attributed to several factors. Initially, during the early stages of the CTP implementation, technological and market immaturity may prevail. Businesses are required to innovate technically to reduce carbon emissions and minimize costs, but this process requires considerable time, resources, and expertise. Consequently, provinces and cities participating in carbon trade trials might experience a drain of talent and resources from neighboring areas, partly due to increased demand for skilled individuals. This, in turn, could impede the ecological development of adjacent regions.

Following the implementation of the CTP in pilot regions, the operational costs of businesses tend to rise, potentially dampening their competitiveness. Meanwhile, surrounding regions not subject to carbon trade policies benefit from lower production costs, resulting in a stronger competitive advantage. This imbalance exacerbates greenhouse gas and atmospheric pollutant emissions. The competitive pressure may lead to the loss of industrial firms and job opportunities in pilot regions, ultimately affecting their ecological development.

Furthermore, businesses in pilot regions may relocate to neighboring areas to avoid the restrictions imposed by the carbon trading policy. This industrial migration could weaken the industrial framework in pilot regions while increasing industrial carbon emissions and atmospheric pollution levels in adjacent areas.

Despite initially potentially restraining the CEE in surrounding areas, in the long run, the CTP is expected to incentivize enterprises to innovate and improve technologies to meet carbon emission quota requirements. Over time, low-pollution technologies and production methods are expected to advance. Moreover, through technology transfer, advanced low-pollution technologies will be disseminated and adopted in surrounding areas, thereby enhancing overall green development in the entire region.

## Conclusion and recommendations

### Conclusion

Most existing studies on Carbon Trading Policy have focused solely on its impact on carbon emissions, neglecting its influence on both carbon emissions and the emission of air pollutants. This study aims to fill this gap by investigating the additional role of the CTP, assessing its contribution to the CEE and air pollutant emissions, and examining the spillover effect of the CTP pilot cities on the CEE in neighboring areas. This research endeavors to provide reference for refining and improving the CTP.

To calculate the CEE for 30 provinces and cities in China from 2005 to 2020, this study employs the Directional SBM Super-Efficiency Model. Utilizing hotspot analysis, the findings indicate that China's CEE hotspot gradually shifts towards the southeast coastal area over time, while the cold spot region moves towards the northwest.

Using the DID model and conducting robustness checks, the study finds that the CTP significantly enhances the CEE development.

Spatial econometric analyses reveal that the CEE predominantly follows LL and HH distribution patterns, exhibiting positive spatial spillover effects. However, due to the early stages of green development and associated imperfections, the CTP temporarily demonstrates negative spatial spillover effects.

### Recommendations

Drawing from the study findings, this paper offers several recommendations.

1. The government should establish comprehensive emission reduction targets encompassing both carbon emissions and air pollutants. This approach ensures that policies address not only greenhouse gas emissions but also air quality improvement.
2. Integrating air pollutant emission rights trading into carbon trading can incentivize enterprises to reduce both carbon and air pollutant emissions. Linking these emissions encourages holistic environmental responsibility among businesses.
3. The government needs to enhance cross-regional policy coordination and integrated planning to ensure consistency in emission reduction policies between pilot cities and neighboring regions. Establishing regional cooperation mechanisms will facilitate joint efforts in emission reduction, fostering cross-border control of emissions and enhancing overall effectiveness.
4. The establishment and enhancement of a cross-regional carbon emissions trading platform are crucial. This platform allows enterprises from pilot cities and neighboring regions to trade carbon emissions collectively, promoting broader emission reduction efforts and effects.
5. Promoting cooperation in environmental protection industries between pilot cities and neighboring regions can drive the development of clean technologies and low-carbon industries. This reduces reliance on high-carbon emission industries, leading to overall reductions in carbon and air pollutant emissions. Additionally, supporting cross-regional emission reduction demonstration projects enables the exploration of effective models and experiences for wider implementation.

## Data Availability

The data supporting the findings of this study are available at: http://meicmodel.org.cn/, http://cnki.nbsti.net/CSYDMirror/trade/yearbook/Single/N2022110021?z=Z005, http://cnki.nbsti.net/CSYDMirror/Trade/yearbook/single/N2022010277?z=Z018, http://cnki.nbsti.net/CSYDMirror/Trade/yearbook/single/N2022060061?z=Z024, https://cnki.nbsti.net/CSYDMirror/Trade/yearbook/single/N2022010064?z=Z016, http://cnki.nbsti.net/CSYDMirror/trade/yearbook/Single/N2022030154?z=Z009, http://cnki.nbsti.net/CSYDMirror/trade/Yearbook/Single/N2022010304?z=Z012, https://data-cnki-net-s.caas.cn/area/Yearbook/Single/N2019030263?dcode=D12.

## References

[1] Ramanathan V, Feng Y (2009). Air pollution, greenhouse gases and climate change: Global and regional perspectives. Atmos. Environ..

[2] Wang R, Zhang Y, Zou C (2022). How does agricultural specialization affect carbon emissions in China?. J. Clean. Prod..

[3] Song Q, Qin M, Wang R, Qi Y (2020). How does the nested structure affect policy innovation? Empirical research on China’s low carbon pilot cities. Energy Policy.

[4] Van Vuuren DP, Cofala J, Eerens HE, Oostenrijk R, Heyes C, Klimont Z, Den Elzen MGJ, Amann M (2006). Exploring the ancillary benefits of the Kyoto Protocol for air pollution in Europe. Energy Policy.

[5] Bayer P, Aklin M (2020). The European Union Emissions Trading System reduced CO_2_ emissions despite low prices. Proc. Natl. Acad. Sci. U. S. A..

[6] Chen X, Lin BQ (2021). Towards carbon neutrality by implementing carbon emissions trading scheme: Policy evaluation in China. Energy Policy.

[7] Gu GT, Zheng HR, Tong LY, Dai YX (2022). Does carbon financial market as an environmental regulation policy tool promote regional energy conservation and emission reduction? Empirical evidence from China. Energy Policy.

[8] Li GM, Zhang WJ (2017). Research on industrial carbon emissions and emissions reduction mechanism in China’s ETS. China Popul. Resour. Environ..

[9] Gao YN, Li M, Xue JJ, Liu Y (2020). Evaluation of effectiveness of China’s carbon emissions trading scheme in carbon mitigation. Energy Econ..

[10] Zhou D, Liu YC (2020). Impact of China’s carbon emission trading policy on the performance of urban carbon emission and its mechanism. China Environ. Sci..

[11] Wang H, Li Y, Lin W, Wei WD (2023). How does digital technology promote carbon emission reduction? Empirical evidence based on e-commerce pilot city policy in China. J. Environ. Manag..

[12] Wang YF, Liu J, Zhao ZH, Ren J, Chen XR (2023). Research on carbon emission reduction effect of China’s regional digital trade under the “double carbon” target– combination of the regulatory role of industrial agglomeration and carbon emissions trading mechanism. J. Clean. Prod..

[13] Wang JN, Ning M, Yan G (2010). Implementing climate-friendly strategy for air pollution prevention and control. China Soft Sci..

[14] Burtraw D, Krupnick A, Palmer K, Paul A, Toman M, Bloyd C (2003). Ancillary benefits of reduced air pollution in the US from moderate greenhouse gas mitigation policies in the electricity sector. J. Environ. Econ. Manag..

[15] Thompson TM, Rausch S, Saari RK, Selin NE (2014). A systems approach to evaluating the air quality co-benefits of US carbon policies. Nat. Clim. Change.

[16] Kaya Y, Yokobori K (1997). Environment, Energy, and Economy: Strategies For Sustainability.

[17] Xu L, Fan MT, Yang LL, Shao S (2021). Heterogeneous green innovations and carbon emission performance: Evidence at China's city level. Energy Econ..

[18] Dong F, Li YF, Zhang XY, Zhu J, Zheng L (2021). How does industrial convergence affect the energy efficiency of manufacturing in newly industrialized countries? Fresh evidence from China. J. Clean. Prod..

[19] Dong F, Li YF, Gao YJ, Zhu J, Qin C, Zhang XY (2022). Energy transition and carbon neutrality: Exploring the non-linear impact of renewable energy development on carbon emission efficiency in developed countries. Resour. Conserv. Recycl..

[20] Zhu B, Zhang M, Zhou Y, Wang P, Sheng JC, He KJ, Wei YM, Xie R (2019). Exploring the effect of industrial structure adjustment on interprovincial green development efficiency in China: A novel integrated approach. Energy Policy.

[21] Miao Z, Chen X, Balezentis T (2021). Improving energy use and mitigating pollutant emissions across "three regions and ten Urban agglomerations": A city-level productivity growth decomposition. Appl. Energy.

[22] Liu Y, Dong F (2021). How technological innovation impacts urban green economy efficiency in emerging economies: A case study of 278 Chinese cities. Resour. Conserv. Recycl..

[23] Tone K (2001). A slacks-based measure of efficiency in data envelope analysis. Eur. J. Oper. Res..

[24] Iftikhar Y, He W, Wang Z (2016). Energy and CO2 emissions efficiency of major economies: A non-parametric analysis. J. Clean. Prod..

[25] Tone K (2002). A slacks-based measure of super-efficiency in data envelopment analysis. Eur. J. Oper. Res..

[26] Xie Z, Wu R, Wang S (2021). How technological progress affects the carbon emissionefficiency? Evidence from national panel quantile regression. J. Clean. Prod..

[27] Oh D (2010). A global Malmquist-Luenberger productivity index. J. Prod. Anal..

[28] Wang Z, Xu X, Zhu Y, Gan T (2020). Evaluation of carbon emission efficiency in China’s airlines. J. Clean. Prod..

[29] Toshiyuki S, Yan Y (2017). Social sustainability measured by intermediate approach for DEA environmental assessment: Chinese regional planning for economic development and pollution prevention. Energy Econ..

[30] Sun C, Liu X, Li A (2018). Measuring unified efficiency of Chinese fossil fuel power plants: intermediate approach combined with group heterogeneity and window analysis. Energy Policy.

[31] Cheng Z, Li L, Liu J, Zhang HM (2018). Total-factor carbon emission efficiency of China’s provincial industrial sector and its dynamic evolution. Renew. Sust. Energy Rev..

[32] Fukuyama HF, Weber WL (2009). A directional slacks-based measure of technical inefficiency. Socio-Econ. Plan. Sci..

[33] Ji SH, Zhu YM, Zhang X (2016). Conducted a study on the improvement effect of industrial agglomeration on resource mismatch. J. China Ind. Econ..

[34] Yu B, Fan CL (2022). Green finance, technological innovation and high-quality economic development. Nanjing Soc. Sci..

[35] Fang JG, Lin FL (2019). Conducted a study on the regional differences and influencing factors of green finance development in China. Wuhan Finance.

[36] Sheng P, Li J, Zhai M (2020). Coupling of economic growth and reduction in carbon emissions at the efficiency level: Evidence from China. Energy.

[37] Wu H (2021). Does internet development improve green total factor energy efficiency? Evidence from China. Energy Policy.

[38] Dong F, Li Y, Qin C, Sun J (2021). How industrial convergence affects regional green development efficiency? Evidence from China. J. Environ. Manag..

[39] Li G, Wei X (2021). Financial development, openness, innovation, carbon emissions, and economic growth in China. Energy Econ..

[40] Chen M, Li ZL, Duan LF (2022). Investigated the spatiotemporal evolution pattern and key driving factors of industrial atmospheric pollutant emissions in the Chengdu-Chongqing region. Res. Environ. Sci..

[41] Zhu R, Zhao R, Sun J (2021). Temporospatial pattern of carbon emission efficiency of China’s energy-intensive industries and its policy implications. J. Clean. Prod..

[42] Thellufsen JZ, Lund H, Sorknase P, Stergaard P, Chang M, Drysdale D, Nielsen S, Djørup SR, Sperling K (2020). Smart energy cities in a 100 renewable energy context. Renew. Sust. Energy Rev..

[43] Yu B, Fang D (2021). Decoupling economic growth from energy-related PM2.5 emissions in China: A GDIM-based indicator decomposition. Ecol. Indic..

[44] Li M, Liu H, Geng GN, Hong CP, Liu F, Song Y, Tong D, Zheng B, Cui HY, Man HY (2017). Anthropogenic emission inventories in China: A review. Natl. Sci. Rev..

[45] Zheng B, Tong D, Li M, Liu F, Hong C, Geng G, Li H, Li X, Peng L, Qi J, Yan L, Zhang Y, Zhao H, Zheng Y, He K, Zhang Q (2018). Trends in China’s anthropogenic emissions since 2010 as the consequence of clean air actions. Atmos. Chem. Phys..

[46] Li M, Zhang Q, Streets DG, He KB, Cheng YF, Emmons LK, Huo H, Kang SC, Lu Z, Shao M, Su H, Yu X, Zhang Y (2014). Mapping Asian anthropogenic emissions of non-methane volatile organic compounds to multiple chemical mechanisms. Atmos. Chem. Phys..

[47] Li M, Zhang Q, Zheng B, Tong D, Lei Y, Liu F, Hong C, Kang S, Yan L, Zhang Y, Bo Y, Su H, Cheng Y, He K (2019). Persistent growth of anthropogenic non-methane volatile organic compound (NMVOC) emissions in China during 1990–2017: Drivers, speciation and ozone formation potential. Atmos. Chem. Phys..

[48] Shao S, Fan MT, Yang LL (2022). Economic structural adjustment, green technological progress, and China's low-carbon transformation: An empirical study based on the perspective of overall technological frontier and spatial spillover effects. Manag. World.

